# Supplementary material for: Comparative Analysis of Six Complete Plastomes of Tripterospermum spp
Source: Int J Mol Sci. 2024 Feb 22;25(5):2534. doi: 10.3390/ijms25052534 (PMC10931592; doi:10.3390/ijms25052534)
Supplement: Supplementary file 1 [file ijms-25-02534-s001.zip › Figure_Table_Tite.pdf]

Supplementary Figure S1. Colinear analysis of the six *Tripterospermum* plastomes using MAUVE software. The annotated rRNA, protein-coding and tRNA genes are shown in red, white and green boxes, respectively.

Supplementary Figure S2. Distribution of simple sequence repeats (SSR) in the six *Tripterospermum* plastomes. (A) Number of SSRs in the LSC, IR and SSC regions. (B) Number of SSRs in the intergenic spacer (IGS), intron sequences, and protein-coding regions (CDS).

Supplementary Figure S3. Types and percentage of repeated sequences in the six *Tripterospermum* plastomes. (A) Percentage of four repeat types. (B) Types and number of long repeats. (C) Number of repeats divided by length.

Supplementary Table S1. Complete plastome features of the six *Tripterospermum* plastomes.

Supplementary Table S2. Codon usage within the protein-coding sequences of the six *Tripterospermum* plastomes.

Supplementary Table S3. Types and number of simple sequence repeats (SSR) in the six *Tripterospermum* plastomes.

Supplementary Table S4. Types and number of repeated sequences in the six *Tripterospermum* plastomes.

Supplementary Table S5. Statistics for length, nucleotide variability of coding region and intergenic regions of the six *Tripterospermum* plastomes.

Supplementary Table S6. GenBank accession numbers of the plastid construction phylogenetic tree of Gentianinae, and an asterisk (*) denote sequences obtained in this study.
