# Supplementary figures and images for: Comparative Analysis of Six Complete Plastomes of Tripterospermum spp
Source: Int J Mol Sci. 2024 Feb 22;25(5):2534. doi: 10.3390/ijms25052534 (PMC10931592; doi:10.3390/ijms25052534)

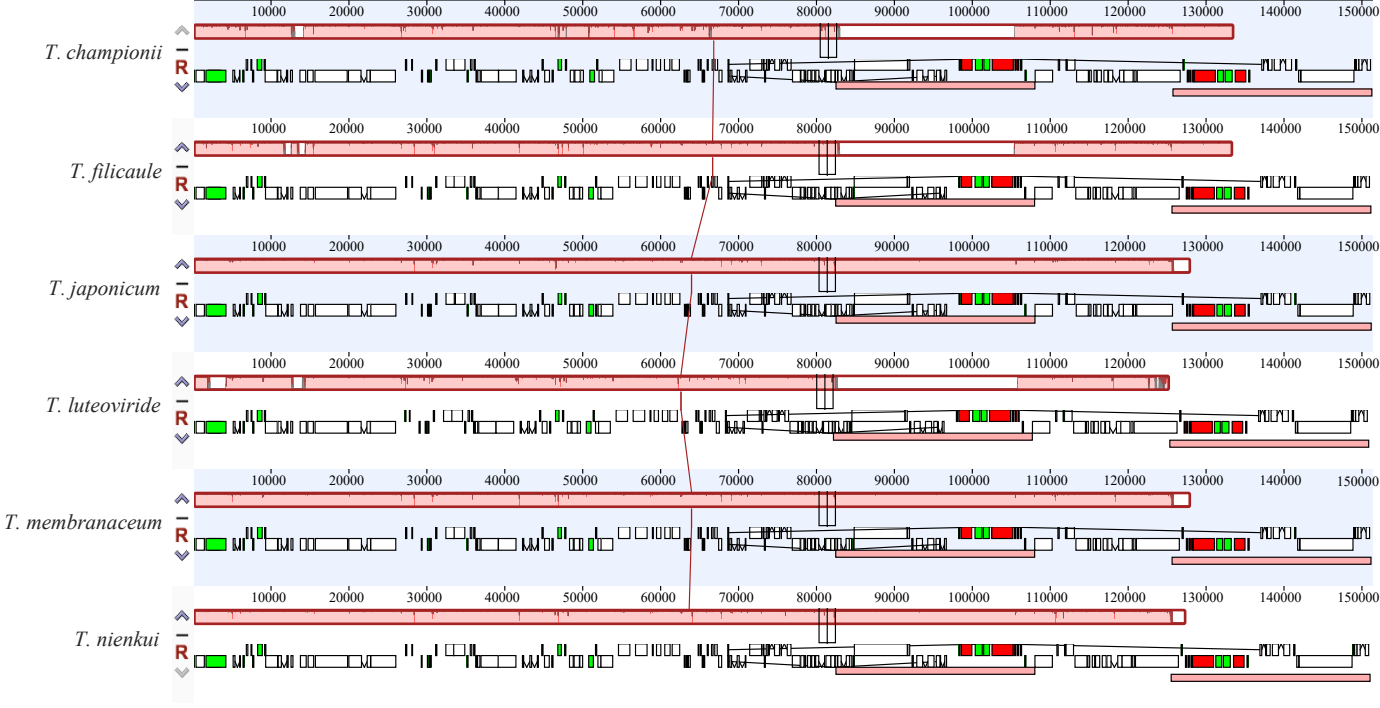

Supplement: Supplementary file 1 [file ijms-25-02534-s001.zip › Supplementary Figure S1.pdf]

**A**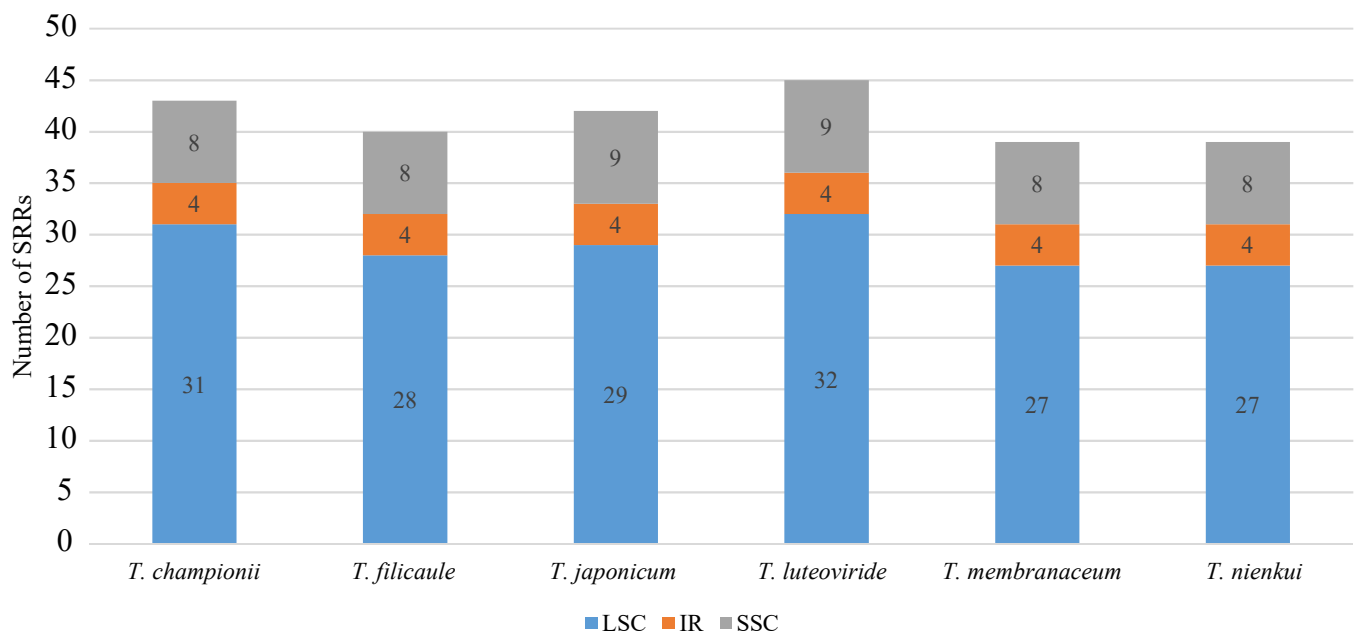**B**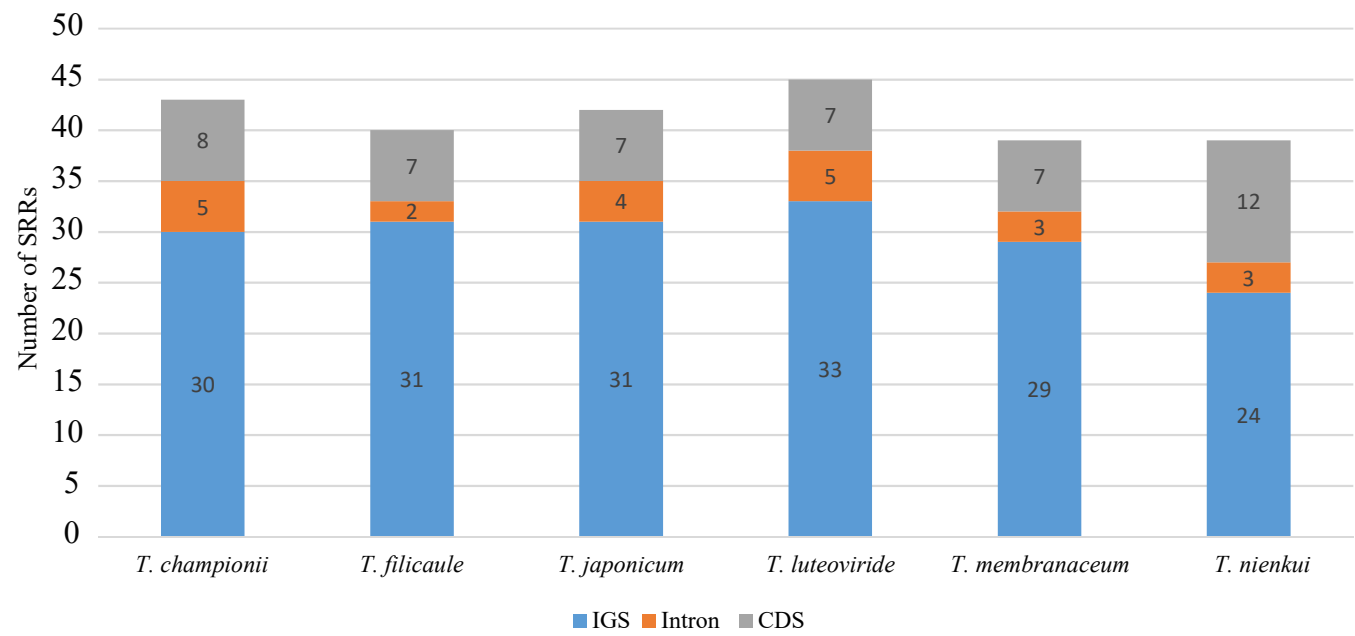

Supplement: Supplementary file 1 [file ijms-25-02534-s001.zip › Supplementary Figure S2.pdf]

A

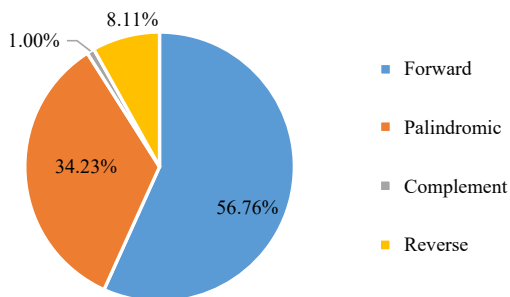

B

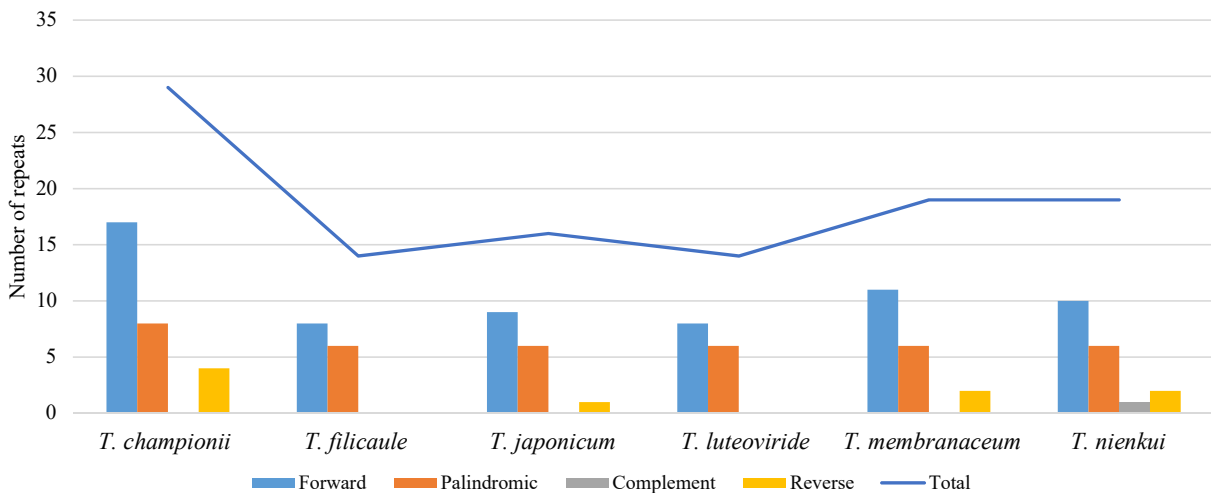

C

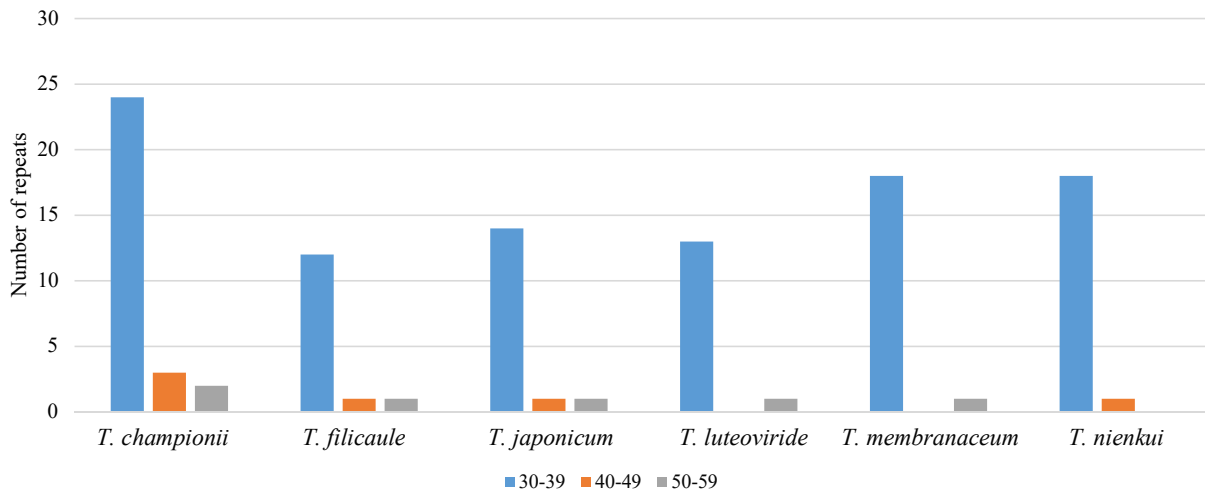

Supplement: Supplementary file 1 [file ijms-25-02534-s001.zip › Supplementary Figure S3.pdf]
